# Closing the Nuclear Fuel Cycle with a Simplified Minor Actinide Lanthanide Separation Process (ALSEP) and Additive Manufacturing

**DOI:** 10.1038/s41598-019-48619-x

**Published:** 2019-09-06

**Authors:** Artem V. Gelis, Peter Kozak, Andrew T. Breshears, M. Alex Brown, Cari Launiere, Emily L. Campbell, Gabriel B. Hall, Tatiana G. Levitskaia, Vanessa E. Holfeltz, Gregg J. Lumetta

**Affiliations:** 1 Radiochemistry Program, Department of Chemistry and Biochemistry, University of Nevada, Las Vegas, NV 89101 USA; 2 Chemical and Fuel Cycle Technology Division, Argonne National Laboratory, Argonne, IL 60439 USA; 3 Nuclear Science Division, Pacific Northwest National Laboratory PO Box 999, Richland, WA 99352 USA

**Keywords:** Nuclear chemistry, Nuclear waste

## Abstract

Expanded low-carbon baseload power production through the use of nuclear fission can be enabled by recycling long-lived actinide isotopes within the nuclear fuel cycle. This approach provides the benefits of (a) more completely utilizing the energy potential of mined uranium, (b) reducing the footprint of nuclear geological repositories, and (c) reducing the time required for the radiotoxicity of the disposed waste to decrease to the level of uranium ore from one hundred thousand years to a few hundred years. A key step in achieving this goal is the separation of long-lived isotopes of americium (Am) and curium (Cm) for recycle into fast reactors. To achieve this goal, a novel process was successfully demonstrated on a laboratory scale using a bank of 1.25-cm centrifugal contactors, fabricated by additive manufacturing, and a simulant containing the major fission product elements. Americium and Cm were separated from the lanthanides with over 99.9% completion. The sum of the impurities of the Am/Cm product stream using the simulated raffinate was found to be 3.2 × 10^−3^ g/L. The process performance was validated using a genuine high burnup used nuclear fuel raffinate in a batch regime. Separation factors of nearly 100 for ^154^Eu over ^241^Am were achieved. All these results indicate the process scalability to an engineering scale.

## Introduction

Nuclear energy is an established and reliable source of electrical power with a greenhouse gas emission footprint comparable to that of wind and photovoltaic solar power^1–3^. A key barrier to expanded use of this power source is the management and disposition of the radioactive by-products of nuclear fission. Currently, there are 98 operating commercial nuclear power reactors in the United States (US) that produce nearly 20% of the electricity in the country^4^. These reactors generate on average 2,000 metric tons (MT) of used nuclear fuel (UNF) annually, and nearly 80,000 MT of UNF has accumulated in the US^4,5^. The current practice of storing this UNF at the power reactor site is not ideal, and the safety of some aspects of this situation has been questioned^6^. At the end of 2016, the US Nuclear Regulatory Committee (NRC) extended a 40-year operating life for 84 reactors built between the year 1970 and 1990^4^. The 20-year license renewal can be repeated once again, increasing the operating life of the light water reactors up to 80 years. If the spent fuel discharge rate remains constant, by 2040 the total US inventory will equal to 126,000 MT. This would exceed the statutory limit for the first US nuclear waste repository by a factor of 2^5^.

Nuclear fuel cycles in which the actinide elements are recycled from UNF offer substantial benefits. An immediate benefit is the recovery and reuse of 95% of the fissionable content the fuel, allowing production of additional power from this resource. Like all recycling activities, this also has upstream environmental benefits by reducing the mining of uranium ore^7^. Furthermore, separation of the so-called minor actinides (MA), including neptunium (Np), americium (Am), and curium (Cm), will minimize the negative downstream impacts of nuclear power^3,8,9^. Following separation, the MAs could be either transmuted to stable or short-lived isotopes in Generation IV fast neutron nuclear reactors, or be disposed of in a compact waste forms specifically designed to sequester these elements^3,9^. The transmutation option offers benefits to geological disposition of UNF by increasing the loading capacity of the repository (by reducing the heat load), and by reducing the long-term radiotoxicity of the disposed material^3^.

To enable this advanced nuclear fuel cycle, cost-effective, high throughput, and reliable separation schemes are needed. Our research has focused on developing simplified methods for separating the MA from UNF, since this is a particularly challenging step in fully closing the nuclear fuel cycle. Furthermore, we have focused our efforts on liquid-liquid extraction methods since this technology is the only industrially proven technique suitable for reprocessing of vast quantities of nuclear materials in a timely manner, and is compatible with current industrial practices^9,10^.

**Figure 1:**
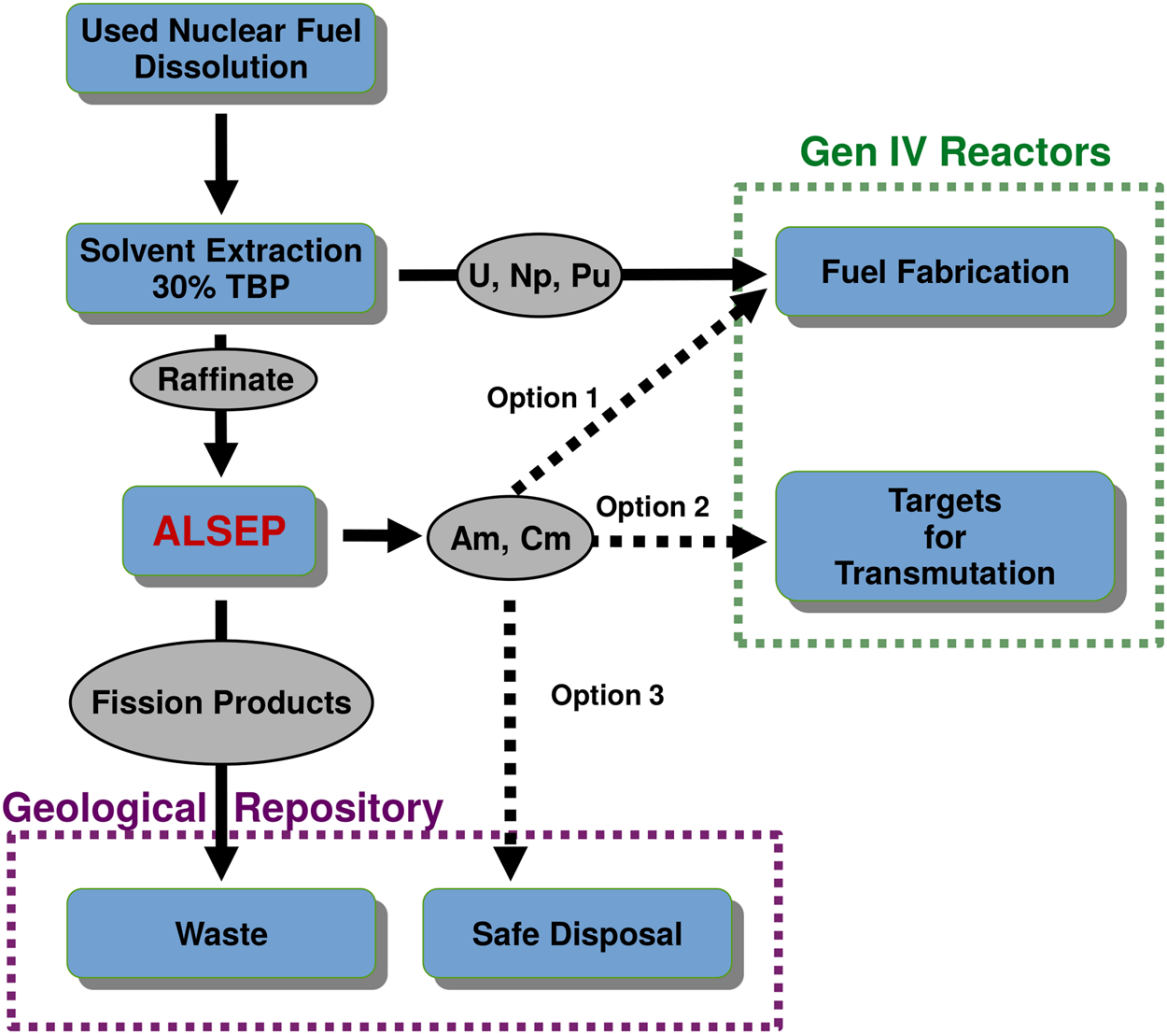
Concept for recovery and recycle of actinide elements in an advanced nuclear fuel cycle.

The starting point is the highly radioactive aqueous solution that is generated after the recovery of uranium, neptunium and plutonium from the dissolved fuel by extraction with tri-butyl phosphate (TBP), such as is practiced in the Plutonium Uranium Reduction Extraction (PUREX) process^3^, or the COEX^™ ^^11^ process. For the MA recovery, the following design criteria were selected for developing a robust and cost-effective process: (1) a single extraction cycle, (2) minimal aqueous feed adjustment for the PUREX raffinate, (3) using conventional industrial chemicals in both the aqueous and organic phases, and (4) radiolytic and hydrolytic stability of the solvent components. Based on these criteria, we have developed the Actinide Lanthanide SEParation (ALSEP) concept (Fig. 1)^12–14^.

## Methods

All chemicals were used as-received, unless otherwise noted. N,N,N′,N′-tetra(2-ethylhexyl)diglycolamide (T2EHDGA) was obtained from Eichrom Technologies at >99% purity. N,N,N′,N′-tetraethyldiglycolamide (TEDGA) was obtained from Technocomm Ltd (Falkland, Scotland, UK). The *n*-dodecane was purchased from Acros Organics at 99% purity. The 2-ethylhexylphosphonic acid mono-2-ethylhexyl ester (HEH[EHP]) was obtained from BOC Sciences, USA, at 95% purity and was purified to 99.5% purity as confirmed by ^31^P NMR spectroscopy, using either the third phase formation purification technique or by the copper precipitation technique^15^. Citric acid and *trans-*1,2-diaminocyclohexane-N,N,N′,N′-tetraacetic acid (CDTA) were received from Sigma Aldrich (ACS reagent grade, >99.5%), while diethylenetriaminepentaacetic acid (DTPA) and N-(2-hydroxyethyl)ethylenediamine-N,N′,N′-triacetic acid (HEDTA) were obtained from Fluka (≥99.0%). Concentrated NH_4_OH (Sigma Aldrich, ACS reagent grade) was used to dissolve the polyaminocarboxylates and adjust pH as checked by a pH electrode (Orion), calibrated with 4.01 and 7.00 pH buffers. All solutions were diluted to the desired volume with deionized water (Millipore, 18.2 MΩ-cm).

Partitioning of metal ions in liquid-liquid extraction is described by the distribution ratio, D, for each metal ion M is defined as the ratio of organic to aqueous metal concentrations or *D* = [M]_org_/[M]_aq_. The separation factor of any two metals, *SF*, is defined as the ratio of their *D* values.

For the equilibrium tracer-level studies, metal extraction was performed by contacting the extracting organic phase with the aqueous phases for 10 minutes using a vortex mixer at the maximum intensity setting, which was found to besufficient to attain equilibrium^13^. Extractions were performed in glass culture tubes followed by centrifugation at 1600_*_g for 5 minutes or until phase disengagement. Phases were separated using a fine tipped transfer pipet.

For the multistage centrifugal contactor test, a simulated PUREX raffinate containing the major fission product elements was prepared in 3 M nitric acid and spiked with approximately 10 microCi/L (3.7 × 10^5^ Bq/L) of ^241^Am, ^244^Cm and ^147^Pm each. The composition of this aqueous feed is based on the ORIGEN 2.1 code. for a light-water reactor fuel burn up of 50,000 MWd/t U with a 5-year cooling period to allow for decay of short lived fission products^16^. The assumed burnup represents the best judgement of technical experts convened by the U.S. Department of Energy Office of Nuclear Energy in a study of nuclear fuel recycling flowsheets (unpublished results). For the run with ^99m^Tc, technetium was stripped from the ^99^Mo/^99m^Tc generator (Lantheus Medical Imaging) with a saline solution using the manufacturer’s instructions.

Microscale extraction experiments were carried out in a pressure-driven slug flow capillary system. The system was fabricated entirely of commercially available components (Dolomite Microfluidics, Ltd.) including a T-junction droplet generator microfluidic chip, 250 µm ID FEP capillary tubing, and a hydrophobic membrane phase separator. Aqueous and organic phase inputs combined at the T-junction into well-defined, reproducible slug flow which was fed into the capillary tubing where extraction occurred until the phases were separated by the in-line phase separator. The final metal ion concentration of the phases was determined by off-line characterization with liquid scintillation counting. The specific interfacial area was measured using image analysis. The method was validated by using the system to measure interfacial mass transfer rate constants for the TALSPEAK solvent extraction process^17^. A full description of the apparatus used in this work was recently reported^18^.

For the tests with the used fuel raffinate, a batch of the irradiated fuel with an average burnup of 60–70,000 MWd/t U was dissolved in 2011. This fuel was irradiated in the Quad Cities-I boiling water reactor. The irradiated fuel was crushed using a commercial tungsten carbide piston-in-cylinder sample crusher. Two approximately 7-g portions of the crushed fuel were dissolved by refluxing for 3 h in 12 mol/L HNO_3_. The undissolved solid phase was separated by centrifugation, then each individual solution was diluted to 200 mL with 2 mol/L HNO_3_. Portions of these solutions (approximately 180 mL each) that were not used for the characterization effort were stored for approximately 5 years. After this time, the solutions were combined and the volume was reduced by evaporation to approximately 110 mL.

To prepare the solution for the used fuel batch ALSEP experiment, the bulk U was removed, along with the Np and Pu, by extraction with tri-butyl phosphate (TBP). A portion (50 mL) of the concentrated dissolved fuel solution was mixed with 24 mL of a solution containing pentavalent vanadium [V(V)] (35 mmol/L) in HNO_3_ (3 mol/L), yielding 11 mmol/L V(V) in the aqueous feed solution. The V(V) was added to allow extraction of Np as Np(VI)^19–21^. The aqueous feed solution was contacted three successive times with fresh 46 mL portions of 30% TBP dissolved in *n-*dodecane. For each contact, the organic and aqueous phases were shaken for 10 minutes in a sealed bottle, and then transferred to a separatory funnel. After the two phases separated, the aqueous phase was drained from the separatory funnel and moved forward to the next contact. This general approach was used for all the batch contacts performed.

Following removal of U, Np, and Pu by TBP extraction, 30 mL of the dissolved fuel solution was mixed with 50 mL of deionized water to adjust the HNO_3_ concentration to 3.5 mol/L. CDTA•H_2_O (1.09 g, 3.00 mmol) was dissolved into 60 mL of the adjusted dissolved fuel solution. The resulting aqueous solution was contacted three successive times with fresh 30 mL portions of the ALSEP solvent (0.05 mol/L T2EHDGA plus 0.5 mol/L HEH[EHP] dissolved in *n-*dodecane). The organic phases from the three ALSEP extraction contacts were combined and scrubbed as follows. The loaded ALSEP solvent (45 mL) was first scrubbed by contacting with 45 mL of 3 mol/L HNO_3_. Two additional scrub steps were performed with an aqueous solution consisting of 1 mol/L acetohydroxamic acid (AHA) plus 0.175 mol/L ammonium citrate at pH 3.3; the organic-to-aqueous phase ratio was 1.0 for each of these scrub contacts. After addition of the AHA solution, the organic phase initially turned purple. However, after equilibration, both phases appeared to be colorless.

The scrubbed, loaded ALSEP solvent was split into two portions. One portion was stripped with 0.015 mol/L DTPA plus 0.175 mol/L ammonium citrate at pH = 2.0 while the other portion was stripped with 0.125 mol/L HEDTA plus 0.2 mol/L ammonium citrate at pH = 3.0. For each MA stripping contact, 20 mL of the loaded solvent was contacted with 30 mL of the DTPA or HEDTA solution. After the first MA stripping contact, the aqueous phase was removed and was replaced with fresh aqueous stripping solution. Finally, the lanthanides were stripped from the solvent by contacting with 0.5 mol/L TEDGA in 1.0 mol/L HNO_3_ at an organic-to-aqueous phase ratio of 0.5. The overall duration of the tests was around 20 hours after the first contact with the feed solution.

For the used fuel raffinate tests the distribution ratios of ^154^Eu and ^241^Am were determined by gamma spectroscopy on a HPGE(Li) gamma-ray detector from the gamma emissions at 123 and 59.5 keV, respectively. The initial concentration of the ^241^Am in the used fuel aqueous feed was found to be 0.18 mM. Liquid scintillation counting on Tri-carb 3100 Packard with α/β discrimination was used to determine the distribution ratios of MA and ^147^Pm for the multistage tests with the simulated PUREX raffinate. ^99m^Tc distribution ratios in a multistage run were determined by gamma counting on a HPGE(Li) detector from the 140.5 keV gamma peak, adjusted for the 6-hour half-life. The distribution ratios for the stable nuclides in the simulated PUREX raffinate or for the selected fuel components in the batch tests were determined by inductively coupled plasma mass spectrometry (ICP-MS). The samples were collected in duplicates or triplicates. The estimated standard deviation did not exceed 3% for all the reported data.

**Figure 2:**
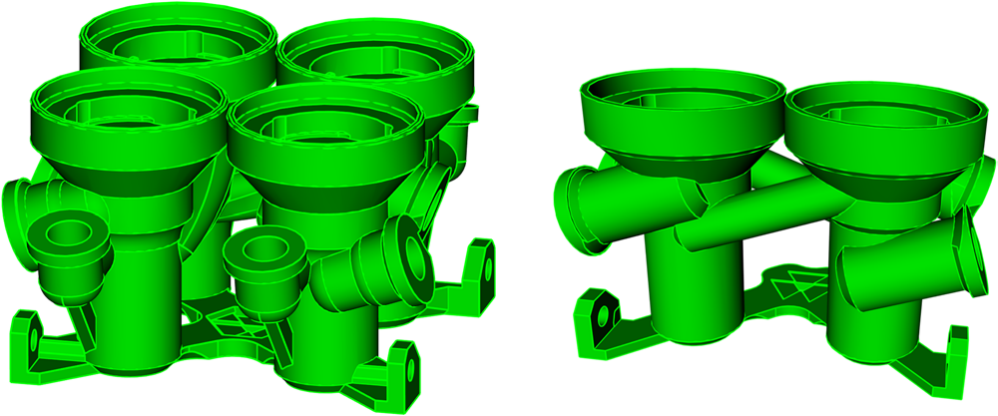
Multi-stage contactor modules used for the development of the ALSEP bank.

Argonne National Laboratory’s additive manufacturing (AM) or 3D printing resources were leveraged to fabricate the contactors for the bench-scale demonstration. AM methods simplify the fabrication process and reduce the cost in terms of human effort and materials (Fig. 2). The layer-by-layer assembly allows for manufacturing of complex fluid devices with internal channels as a single component. Leveraging the flexibility, multiple contactor stages were integrated into single multi-stage modules, reducing the effort required for installation and eliminating potential failure points. Furthermore, multi-stage contactor modules provided flexibility allowing stages to be easily added or removed as the flowsheet was refined.

Except for the electrical components such as the motor and wiring, virtually every part of the contactor bank was 3D printed. The contactor housings, rotors of 1.25-cm diameter, and inter-stage lines were fabricated via stereolithography assembly (SLA) from acrylic. Structural components were printed from carbon-fiber reinforced polyethylene terephthalate on a filament-based printer. The materials were chosen due to their chemical resistance properties along with their mechanical strength. Some post-processing was required for the SLA fabricated parts, in order to ensure the rotors were properly balanced and to reduce wear on the interface between the motor assembly and the housings. The 3.3 V motors installed spun at 1800RPM, allowing for a maximum cumulative counter-current flow rate of 2 mL per minute. Pumping of solutions was accomplished using a set of multi-channel peristaltic pumps and a two-channel syringe pump. For demonstrations requiring a shorter run-time, the higher-accuracy Harvard Apparatus Elite 11 syringe pump was preferred for the feed solution while the peristaltic pumps were ideal for longer run-times.

Argonne Model for Universal Solvent Extraction (AMUSE)^22,23^ was used to generate the ALSEP flowsheet.

## Results

### ALSEP Chemistry

**Figure 3:**
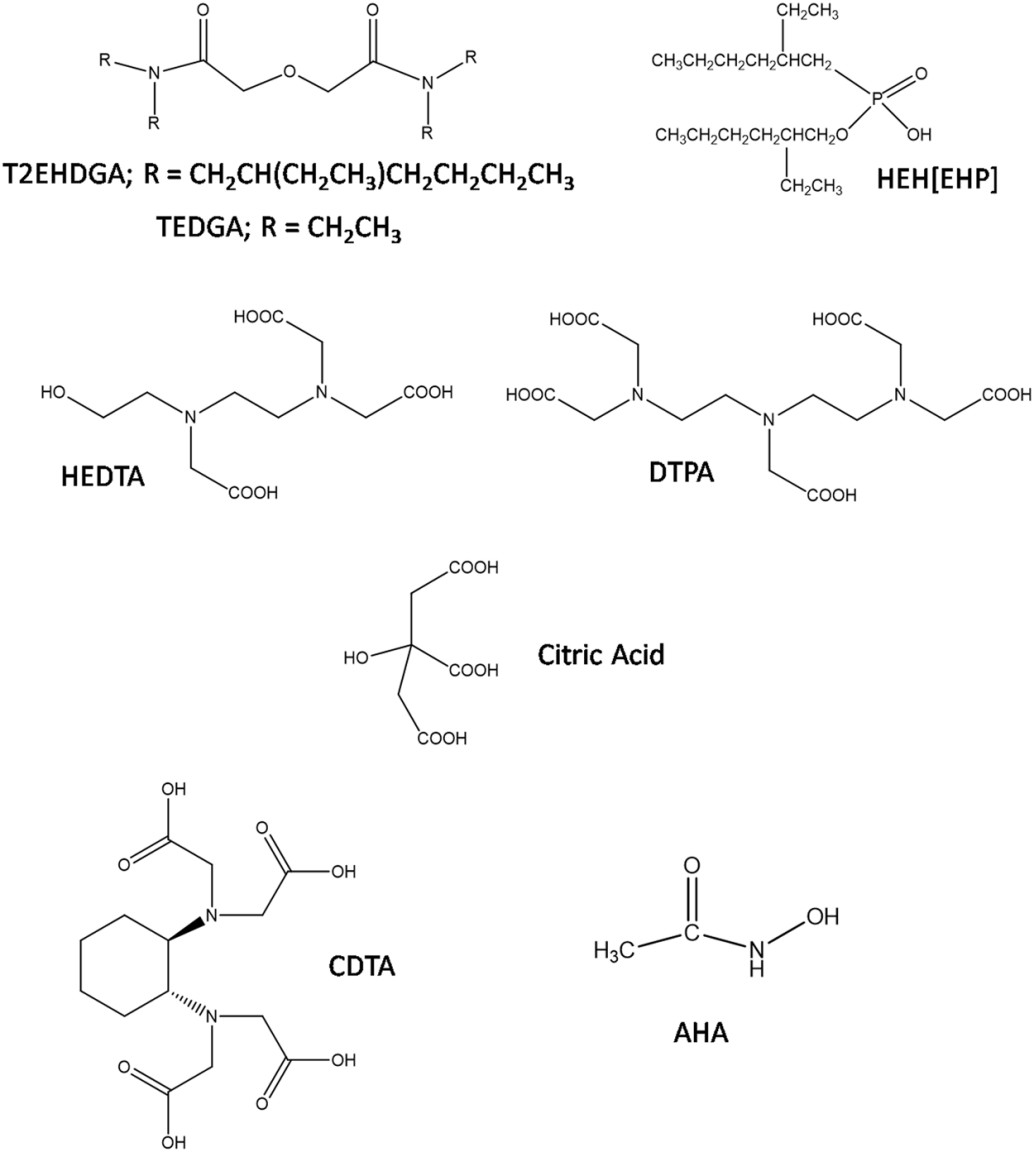
Chemical structures of the extractants and complexants of the ALSEP process.

The ALSEP process utilizes a combination of a neutral extractant, T2EHDGA and an acidic extractant HEH[EHP], dissolved in an aliphatic diluent (e.g., *n*-dodecane) (structures are shown in Fig. 3). A detailed description of the chemistry of the ALSEP process has been previously reported^12–14^. In general, there are two operational acidic regimes of the process: moderately acidic and low acidic. In the moderately acidic regime, T2EHDGA extracts MAs from aqueous solutions that contain several mole/L HNO_3_. The trivalent Ln elements with Z ≥ 58 (cerium and above) are also extracted by T2EHDGA; La is only weakly extracted with *D*_*La*_ values < 1 for the HNO_3_ concentrations of practical importance. The extraction of the trivalent f-block elements is described by Reaction 1.

1 $${{\rm{M}}}^{3+}+3{{{\rm{NO}}}_{3}}^{-}+3{\rm{T}}2{{\rm{EHDGA}}}_{{\rm{org}}}\iff {({\rm{M}}{({{\rm{NO}}}_{3})}_{3}{{\rm{T2EHDGA}}}_{3})}_{{\rm{org}}}$$

The acidic extractant, HEH[EHP], exists in *n-*dodecane as a dimer, presented for simplicity as H_2_L_2_. The HEH[EHP] does not extract the trivalent cations from HNO_3_ of moderate acidity^24,25^. However, there is evidence that it significantly improves the solubility of the Ln(NO_3_)_3_T2EHDGA_3_ complexes in the organic phase thus preventing the formation of a metal-loaded heavy organic layer^12,14^.

During the following step, the organic phase is contacted with a low acidity aqueous solution (i.e., the low acidity regime), resulting in a halfway deprotonation of the HEH[EHP] dimer^24,25^. The function of the HEH[EHP] is to complex and hold the Ln ions in the organic phase, while the MAs are selectively stripped into a buffered solution containing a polyaminocarboxyllateligand. The formation of the lipophilic Ln complexes with HEH[EHP] is governed by Reaction 2.

2 $${({\rm{M}}{({{\rm{NO}}}_{3})}_{3}{({\rm{T}}2{\rm{EHDGA}})}_{3})}_{{\rm{org}}}+3{{\rm{H}}}_{2}{{\rm{L}}}_{2{\rm{org}}}\iff {\rm{M}}{({{\rm{HL}}}_{2})}_{3{\rm{org}}}+3{{\rm{HNO}}}_{3}+3{\rm{T}}2{{\rm{EHDGA}}}_{{\rm{org}}}$$

The aqueous solution chemistry for selectively stripping the MAs from the loaded solvent is based on the aqueous-phase complexation of the MA ions by a polyaminocarboxylate ligand. The soft base character of the amine groups is believed to lead to preferential binding of the polyaminocarboxylate to the actinide ions, which have slightly softer acidic character than the Ln^26^. Two polyaminocarboxylate ligands were investigated for application in the ALSEP process: DTPA and HEDTA. While most of the Ln remain strongly bound to HEH[EHP], the MAs are backward-extracted with a deprotonated soft-donor ligand, DTPA^5−^ or HEDTA^3−^, according to Reaction 3^27^.

3 $${{{\rm{MDTPA}}}^{2-}}_{{\rm{aq}}}+3{{\rm{H}}}_{2}{{\rm{L}}}_{2{\rm{org}}}\iff {\rm{M}}{({{\rm{HL}}}_{2})}_{3{\rm{org}}}+{{\rm{H}}}_{3}{{{\rm{DTPA}}}^{2-}}_{{\rm{aq}}}$$

The ALSEP backward-extraction stripping solution is buffered to control the pH similar to TALSPEAK and an advanced TALSPEAK processes^28,29^. Ammonium citrate buffer has been used in the recent ALSEP process studies. Besides the pH control, the buffer has also been shown to improve the reaction kinetics^28^. The mechanism of the interfacial mass-transfer for TALSPEAK, studied at the constant hydrogen ion concentration of 1 mmol/L, was reported to have multiple steps involving complex formation between cations and carboxylate anions at the interface^30^. In general, the slow extraction rates may be counterbalanced by using modified centrifugal contactors with extended mixing zone that results in prolonged residence time^31^. However, longer residence time of the feed streams decreases the throughput of the process, which in turn would affect the cost-effectiveness of the overall advanced UNF cycle. Thus, the preferred approach is to choose conditions that would allow selective stripping of the MAs from the ALSEP solvent at a practical rate.

### Microfluidic kinetic studies

The original ALSEP solvent formulation was 0.05 mol/L T2EHDGA and 0.75 mol/L HEH[EHP], dissolved in *n-*dodecane^13,14^. However, the Am backward-extraction rates with either DTPA or HEDTA were low enough to cause inefficient stripping of the MA when standard industrial centrifugal contactors with approximately 20 s residence time were used^31^. To optimize the process for high throughput centrifugal contactors, we have applied previously described droplet-based microfluidic method to study the solvent extraction kinetics^17,18^. Advantages of this microscale method include: excellent contact-time temporal resolution, rapid intra-phase mixing, and well-defined specific interfacial areas. The flow rates and capillary tubing dimensions were optimized to achieve the required contact times while minimizing thin film participation in phase transfer, and while generating sufficient intra-phase mixing such that extraction took place in the kinetic regime where the effects of diffusion rates are negligible^17^.

**Figure 4:**
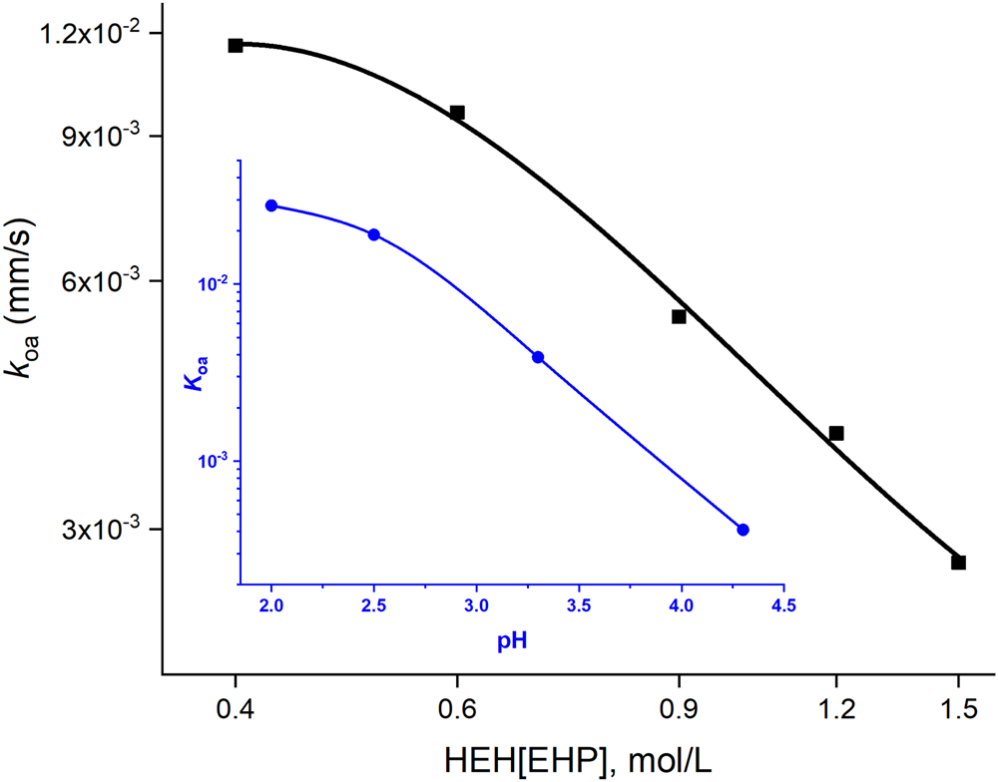
Am backward-extraction rate constant as a function of the HEH[EHP] concentration and the pH of the DTPA buffered solution (inset). Aqueous phase - 0.015 mol/L DTPA, 0.2 mol/L (H/NH_4_)_3_Citrate, pH 2.0; variable HEH[EHP] conc.; Inset - 0.75 mol/L HEH[EHP]/n-ddn; Aqueous phase - 25 mmol/L DTPA, 0.5 mol/L (H/NH_4_)_3_ Citrate, variable pH.

For the partitioning of Am according to Reaction 3, the interfacial mass-transfer was treated as pseudo-first order reaction and the backward-extraction rate constant, *k*_oa_ was measured as the function of the pH and the concentration of HEH[EHP] (Fig. 4).

The kinetic results in Fig. 4 demonstrate that the Am backward-extraction kinetics strongly depends on the pH of the aqueous phase and the concentration of the acidic extractant. The Am stripping step should be conducted at the lowest pH and the lowest concentration of HEH[EHP] that could provide sufficient Ln/MA separation. The Ln/MA selectivity of DTPA drops sharply below pH 1.8^28^, thus the pH in the 1.9–2.3 range was selected for the process application. The corresponding selectivity of HEDTA decreases below pH of 2.5^24^, making HEDTA usable for MA recovery in the ALSEP process only if longer than 20 s contact time is achievable, or higher pH conditions are employed^31^.

Therefore, while the T2EHDGA concentration was kept at 0.05 mol/L, the concentration of HEH[EHP] was lowered to 0.5 mol/L in *n*-dodecane. This solvent formulation in combination with 0.015 mol/L DTPA/0.2 mol/L (H/NH_4_)_3_Citrate, pH 2.0 aqueous solution, provides sufficiently low distribution ratio for Am about 0.1, while the worse-case separation factor (Pm/Am) reaches 30 at equilibrium as it was demonstrated in a separate experimental run using culture tubes and single stage contactor units.

### The ALSEP flowsheet

**Figure 5:**
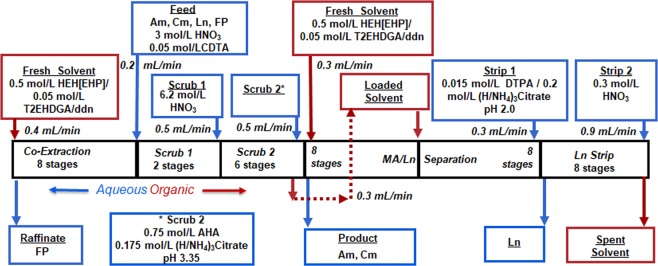
ALSEP countercurrent flowsheet.

The flowsheet shown in Fig. 5 was developed using the AMUSE code and equilibrium experimental data points for partitioning of MA and major fission products^13,31^. The number of stages in each section was calculated using the experimental distribution ratios to provide >99.9% recovery of the minor actinide from the feed and >99.9% MA/Ln separation. The flowsheet consists of 8 extraction stages in which the Ln and MA are co-extracted from the aqueous HNO_3_ (3 mol/L) feed solution into the ALSEP solvent phase. Zirconium is known to exhibit insolubility and undesired solvent extraction behavior and consequently, to prevent fission product Zr from extracting, CDTA (Fig. 3) is added to the aqueous ALSEP feed solution as a masking agent^32^. The loaded solvent flows into a series of two scrub sections. The first scrub (6.2 mol/L HNO_3_) is designed to maintain the acidity in the co-extraction section. The second scrub uses a citrate-buffered AHA solution. This serves two functions. First, the AHA strips fission product Mo (which is strongly extracted from the HNO_3_ feed) from the loaded solvent. Second, the citrate buffer removes much of the residual HNO_3_ from the loaded ALSEP solvent so that it does not interfere in the MA stripping step.

In order to accomplish a complete separation of the MA from the lanthanides, the loaded solvent after Scrub 2 section is routed to the central stage of the MA/Ln separation section, while a stream of the fresh ALSEP solvent is fed to the first stage of the section. This fresh solvent stream serves to “re-extract” any Ln that partitions to the aqueous phase by during MA stripping by the citrate-buffered DTPA solution. The final section of the flowsheet uses dilute HNO_3_ to strip the remaining Ln from the ALSEP solvent.

### Multistage demonstration results

**Figure 6:**
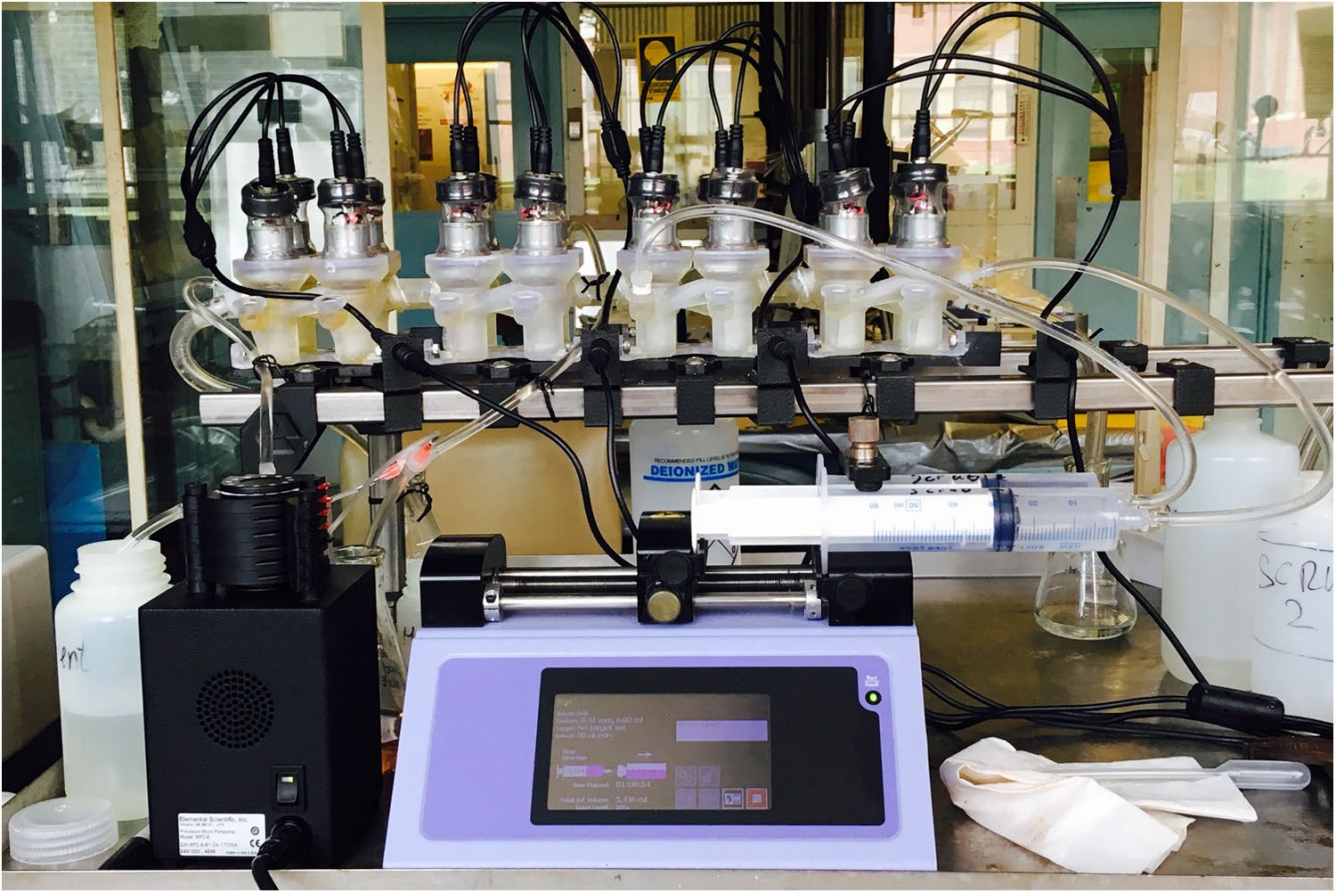
ALSEP demonstration experimental setup.

The flowsheet shown in Fig. 5 was tested over a period of three days using a setup shown in Fig. 6. The co-extraction and scrubbing steps were performed on the first day; the MA stripping section was performed on the following day; and the Ln stripping section was performed on the last day. Timed samples of each process stream including raffinate, solvent and aqueous strips were collected upon reaching steady-state. The time required for a contactor array to reach steady-state can be solved for by reducing the array to a series of simple mixing tanks. Each section (extraction, scrub, strip) contains a total holdup volume, *V*_*section*_, and the sum of the throughputs passing through the section, *q*_*section*_. Assuming ideal mixing within the annular zone, the concentration within each section can be modeled as a differential equation^33^, which is then solved to be Eq. 4 (see Supplementary Information):

4 $${C}_{section}=({C}_{section,0}-{C}_{i}){e}^{-\frac{{q}_{\text{sec}tion}}{{V}_{\text{sec}tion}}t}+{C}_{i}.$$

While the concentration within the section is not of particular interest, the time constant, $\tau =\frac{{V}_{section}}{{q}_{section}}$, can be used to approximate the time to steady-state. For the contactor demonstrations, the runtime was chosen to be ${t}_{ss}=3\tau$, which brings the contactor performance to within 5% of steady-state.

After collecting the time samples, the process was run to accumulate a sufficient amount of the organic phase to be used in the test of the next section of the flowsheet. Table 1 presents the composition of the process streams generated during the demonstration.

**Table 1:** Composition of the ALSEP streams under the steady-state.

Element	Raffinate µg/L	Raffinate %	MA product* µg/L	MA product %	Ln product µg/L	Ln product %	Spent solvent %
Sr	3.93E + 04	98.31	1.37E + 02	0.15	1.94E + 01	0.06	1.47
Y	6.34E + 00	0.03	8.60E − 02	0.00	1.51E + 03	8.30	91.67
Zr	1.16E + 05	86.52	1.04E + 02	0.03	1.09E + 01	0.01	13.44
Mo	8.67E + 04	102.16	2.17E + 03	1.12	2.41E + 01	0.04	0.00
Ru	5.50E + 04	98.14	1.39E + 02	0.11	1.78E + 01	0.04	1.71
Rh	1.79E + 02	101.61	5.96E − 01	0.15	4.16E − 01	0.30	0.00
Pd	2.78E + 03	100.94	3.17E + 00	0.05	3.37E + 00	0.16	0.00
Sn	2.04E + 03	105.34	2.05E + 01	0.46	4.36E + 00	0.28	0.00
Te	1.24E + 04	91.96	1.69E + 01	0.06	1.81E + 01	0.17	7.81
Cs	1.25E + 05	96.82	1.38E + 02	0.05	3.77E + 01	0.04	3.09
La	7.15E + 04	99.80	1.51E + 02	0.11	9.94E + 02	2.08	0.00
Ce	6.18E + 04	53.50	1.33E + 02	0.05	1.36E + 04	14.97	31.48
Pr	1.77E + 03	3.48	4.90E + 01	0.04	1.71E + 04	42.75	53.73
Nd	1.15E + 00	0.00	1.26E + 02	0.03	1.09E + 05	70.37	29.60
Sm	1.31E + 01	0.03	5.03E + 00	0.01	3.43E + 04	109.14	0.00
Eu	1.99E + 00	0.02	7.95E − 01	0.00	6.89E + 03	103.90	0.00
Gd	5.55E + 02	5.40	3.38E + 00	0.01	6.86E + 03	84.95	9.63
^147^Pm		ND		<0.05		>99.95	ND
MA (Am + Cm)		ND		>99.95		<0.05	ND

(Percentage is given relative to the feed considering the dilution factors. ND- not detected). *SUM = 3.2 mg/L.

**Figure 7:**
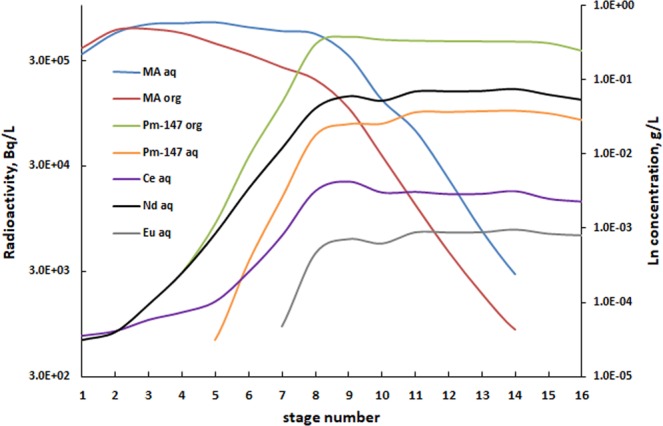
Stage concentrations of the ALSEP MA/Ln separation section. MA (Am + Cm) and Pm concentrations are given in Bq/L; stable Ln (Ce, Nd and Eu) are given in g/L.

The stage samples of the MA/Ln section were collected at the end of the process run, and the phases were separated from each other and analyzed by LSC (organic samples) and LSC and ICP-MS (aqueous samples). The stage profile of the MA/Ln separation section is presented in Fig. 7.

A separate run was conducted to study *technetium* behavior in the co-extraction and 2 scrub sections (Fig. 5). A spike of ^99m^Tc was added to the feed containing Am, Cm and Pm. The flowsheet was modified so there were 6 stages in co-extraction, 2 stages in Scrub 1 and 8 stages in Scrub 2 stages. Upon reaching the steady-state conditions, time samples were collected and analyzed for Tc content. More than 99.9% of Tc resided in the raffinate.

### Used fuel batch results

Table 2 presents the distribution ratios for ^154^Eu and ^241^Am and for selected other fuel components measured during the batch ALSEP test with dissolved irradiated fuel. The distribution ratios for the tri-valent f-block elements Am, Ce, Nd, and Eu during the extraction steps were all consistent with previous extraction studies of these elements in the ALSEP system. The distribution ratios for these lanthanide and actinide elements were greater than 1, resulting in their co-extraction from the HNO_3_-based feed solution. The distribution ratios for these elements remained high during the scrubbing steps, indicating their retention in the organic phase during scrubbing. Both the HEDTA and DTPA stripping solutions were very effective at stripping Am from the loaded ALSEP solvent. The Am distribution ratios were 0.1 to 0.2 under the stripping conditions employed, whereas those for Ce, Nd, and Eu remained well above 1. The Eu was essentially quantitatively stripped from the ALSEP solvent by contact with 0.5 mol/L TEDGA in 1.0 mol/L HNO_3_. However, the Ce and Nd distribution ratios during the TEDGA contact were ~1, indicated further optimization of the lanthanide stripping step is required.

**Table 2:** Distribution ratios for various fuel components in the batch ALSEP experiment with dissolved irradiated fuel raffinate.

Process Step	^154^Eu	^241^Am	Ce	Nd	Mo
ALSEP Extraction 1	13.9	3.3	1.9	5.6	310
ALSEP Extraction 2	(a)	(a)	5.4	17	12
ALSEP Extraction 3	(a)	(a)	3.5	11	0.7
3 mol/L HNO_3_ scrub	59.3	10.5	4.7	16	430
AHA Scrub 1	315	20.3	80	110	69
AHA Scrub 2	(a)	31.2	47	73	0.05
HEDTA MA^(b)^ Strip 1	10.4	0.1	9.1	4.6	19
HEDTA MA Strip 2	10.9	0.2	16	9.4	40
Post-HEDTA Ln Strip 1	(c)	(c)	1.0	1.3	8.5
DTPA MA Strip 1	9.9	0.1	5.8	3.2	3.9
DTPA MA Strip 2	8.7	0.1	7.8	4.4	12
Post-DTPA Ln Strip 1	(c)	(c)	1.3	1.1	38

^(a)^No counts detected in the aqueous phase.

^(b)^MA = minor actinide (i.e., Am and Cm).

^(c)^No counts detected in the organic phase.

## Discussion

Separating the Ln from the MA on a large-scale, continuous basis is a particularly challenging concept since both sets of elements exhibit a stable trivalent oxidation state in aqueous solution and similar solvent extraction profiles. However, owing to the larger 5-*f* electron orbitals, there is an increased covalent bonding nature within the actinides compared to the lanthanide 4-*f* series^26,34^. The separation chemistry designed within ALSEP exploits the covalency of Am^3+^ and Cm^3+^ (which exhibit more covalent bonding characteristics than similar sized Nd^3+^ and Pm^3+^) by using soft-donor nitrogen-rich water-soluble aminopolycarboxylates^26^. Although the increase in bonding equilibria is only roughly one order of magnitude, optimizing the process conditions with respects to extraction chemistry and the stage efficiency, we have developed and demonstrated a flowsheet capable of treating large inputs of a nitric acid stream that contains Ln, MA, and transition metal fission fragments in a single step.

An alternative approach to the MA/Ln separation from PUREX raffinate involves utilizing soft-donor ligands soluble in the organic phase^8,10,35,36^. In this system, MA are preferentially complexed over Ln by the hydrophobic organic ligand, while the Ln remain in the aqueous phase. However, these soft-donor extractants tend to interact very strongly with such transition metals as Pd, Tc, Ru and Rh, present in the used fuel raffinate in ample quantities, resulting in contamination of the MA product stream^37^.

For the first time, additive manufacturing was utilized for fabricating a full array of 1.25-cm rotor diameter centrifugal contactors. This allowed for demonstrating the ALSEP process using very small volumes of the process solutions and, consequently, small quantities of radionuclides. Based upon the results of the microfluidic kinetic studies, the ALSEP process conditions were modified to overcome the previously reported kinetic issues. The modified ALSEP flowsheet was tested in the bank of 1.25-cm centrifugal contactors. Because the performance of centrifugal contactors scales well with increasing rotor diameter, successful implementation with 1.25-cm centrifugal contactors would be expected to translate to successful implementation in larger, industrially-relevant, contactors^38^.

According to Table 1, the MAs were completely extracted from the feed during the countercurrent flowsheet experiment and quantitatively (>99.95%) transferred to the MA product stream. Less than 0.5 mg/L of total Ln was found in the MA product, and no actinides were detected in the Ln product. The measured separation factors (SF) for the Pm/MA (worst case scenario) were in the range of 17 to 24, which is consistent with what would be expected from batch the equilibrium distribution ratio values. Overall decontamination factors over 1000 were accomplished for the MA/Ln separation.

The Am concentration in the first 3 organic stage samples (Fig. 7) is close to the Am concentration in the aqueous stage samples, indicating 50/50 partitioning of Am in these stages. We measured the pH of the aqueous samples and found that for stages 1 through 8 the pH is lower by 0.2–0.3 units than for the stages 9 through 16. It is likely caused by partitioning of hydrogen ions during the contact of the aqueous phase with the fresh solvent in the first half of the contactor bank. As the pH decreases to 1.7, the Am and the Ln distribution ratios increase as the complexing power of DTPA is decreasing. As a positive outcome, the Ln were completely removed from the aqueous phase. Therefore, this pH shift promotes the overall MA/Ln separation. It is important to note that the ALSEP pH profile (D values plotted vs. pH) with the citrate buffer resembles closely the flat pH profile of the advanced TALSPEAK process^13,24^. The original TALSPEAK process demonstrated a rather steep pH profile, which is believed to be unsuitable for process applications^27,28^.

Using the mass balance, the residual concentrations of the FP were calculated for the spent solvent stream (Table 1). According to the calculations, most of the yttrium, about 40% of the light Ln and 13% of Zr remained in the organic phase. While the Y and Zr results are consistent with our equilibrium data, Ce-Nd recovery appears to be low. Surprisingly, Pm was completely stripped while 30% of Nd, which should behave similarly, remained in the organic phase. The stripping and the solvent scrub conditions will need to be improved to completely remove Y, Zr and the Ln from the ALSEP solvent so that it can be recycled in the process. Well known reagents such as oxalic acid and 2-hydroxyethyl diphosphonic acid^39^ are reasonable candidates for use in solvent cleanup.

The countercurrent ALSEP flowsheet experiments demonstrated that the ALSEP process provides very effective means for separation of MA from the fission products after U/Np/Pu removal by TBP. The ALSEP process chemistry was further verified by the results of the batch experiment performed with dissolved UNF. The latter experiment also proved the ALSEP chemistry is robust in a harsh radioactive environment—an essential requirement for ultimate implementation of such a process. Taken together, this work opens the door for a simplified means to recycle the transuranic actinide elements in the nuclear fuel cycle, helping to enable the expansion of low-carbon nuclear power production.

## Supplementary information

Supplementary inforamtion
