# Author Correction: Closing the Nuclear Fuel Cycle with a Simplified Minor Actinide Lanthanide Separation Process (ALSEP) and Additive Manufacturing

**DOI:** 10.1038/s41598-020-57574-x

**Published:** 2020-01-14

**Authors:** Artem V. Gelis, Peter Kozak, Andrew T. Breshears, M. Alex Brown, Cari Launiere, Emily L. Campbell, Gabriel B. Hall, Tatiana G. Levitskaia, Vanessa E. Holfeltz, Gregg J. Lumetta

**Affiliations:** 1 Radiochemistry Program, Department of Chemistry and Biochemistry, University of Nevada, Las Vegas, NV 89101 USA; 2 Chemical and Fuel Cycle Technology Division, Argonne National Laboratory, Argonne, IL 60439 USA; 3 Nuclear Science Division, Pacific Northwest National Laboratory, PO Box 999, Richland, WA 99352 USA

Correction to: *Scientific Reports* 10.1038/s41598-019-48619-x, published online 06 September 2019

Some of the methodology presented in this Article was published previously. The Authors neglected to cite this, which is included below as Reference 1 and should be cited in the Methods section, in the paragraph below figure 2:

“Leveraging the flexibility, multiple contactor stages were integrated into single multi-stage modules, reducing the effort required for installation and eliminating potential failure points”

should read:

“Leveraging the flexibility, multiple contactor stages were integrated into single multi-stage modules, reducing the effort required for installation and eliminating potential failure points^1^”

Reference

1. Wardle, K. E. 3D printed modular centrifugal contactors and method for separating moieties using 3D printed optimized surfaces, US Patent 9,744,476 (Publication date 29-08-2017).

Additionally, the acknowledgements section in this Article is incomplete:

“This work was supported by the U.S. Department of Energy, Office of Nuclear Energy, Nuclear Technology Research and Development Program under Contract DE-AC02-06CH11357. The submitted manuscript has been partially created by UChicago Argonne, LLC, Operator of Argonne National Laboratory (“Argonne”). Argonne, a U.S. Department of Energy Office of Science laboratory, is operated under Contract No. DE-AC02-06CH11357. The U.S. Government retains for itself, and others acting on its behalf, a paid-up nonexclusive, irrevocable worldwide license in said article to reproduce, prepare derivative works, distribute copies to the public, and perform publicly and display publicly, by or on behalf of the Government. Pacific Northwest National Laboratory is operated by Battelle Memorial Institute for the U.S. Department of Energy under contract DE-AC05-76RL01830. We thank Dr. Candido Pereira (ANL, USA), Dr. Giuseppe Modolo (Forschungszentrum Jülich, Germany), Dr. Andreas Geist (Karlsruhe Institute of Technology, Germany) and Dr. Stephan Bourg (CEA-France) for fruitful discussion.”

should read:

“This work was supported by the U.S. Department of Energy, Office of Nuclear Energy, Nuclear Technology Research and Development Program under Contract DE-AC02-06CH11357. The submitted manuscript has been partially created by UChicago Argonne, LLC, Operator of Argonne National Laboratory (“Argonne”). Argonne, a U.S. Department of Energy Office of Science laboratory, is operated under Contract No. DE-AC02-06CH11357. The U.S. Government retains for itself, and others acting on its behalf, a paid-up nonexclusive, irrevocable worldwide license in said article to reproduce, prepare derivative works, distribute copies to the public, and perform publicly and display publicly, by or on behalf of the Government. Pacific Northwest National Laboratory is operated by Battelle Memorial Institute for the U.S. Department of Energy under contract DE-AC05-76RL01830. We thank Dr. Candido Pereira (ANL, USA), Dr. Giuseppe Modolo (Forschungszentrum Jülich, Germany), Dr. Andreas Geist (Karlsruhe Institute of Technology, Germany) and Dr. Stephan Bourg (CEA-France) for fruitful discussion. The authors also thank Dr. Kent Wardle (TerraPower, USA) for his work in generating the initial AM centrifugal contactor designs and prototypes.”

