# Supplementary material for: Closing the Nuclear Fuel Cycle with a Simplified Minor Actinide Lanthanide Separation Process (ALSEP) and Additive Manufacturing
Source: Sci Rep. 2019 Sep 6;9:12842. doi: 10.1038/s41598-019-48619-x (PMC6731290; doi:10.1038/s41598-019-48619-x)
Supplement: Supplementary file 1 — Supplementary inforamtion [file 41598_2019_48619_MOESM1_ESM.pdf]

Supplementary Information

Closing The Nuclear Fuel Cycle With A Simplified Minor Actinide Lanthanide Separation Process (ALSEP) And Additive Manufacturing.

Artem V. Gelis[1*], Peter Kozak[2], Andrew T. Breshears[2], M. Alex Brown[2], Cari Launiere[2], Emily L. Campbell[3], Gabriel B. Hall[3], Tatiana G. Levitskaia[3], Vanessa E. Holfeltz[3], Gregg J. Lumetta[3**]

Affiliations:

[1] Radiochemistry Program, Department of Chemistry and Biochemistry, University of Nevada, Las Vegas, NV, 89101

[2] Chemical and Fuel Cycle Technology Division, Argonne National Laboratory, Argonne, IL 60439

[3]Nuclear Science Division, Pacific Northwest National Laboratory PO Box 999, Richland, WA 99352

* artem.gelis@unlv.edu , **gregglumetta@pnnl.gov

Calculation of the Transient Settling Time for a CCS Section

A simple, first-order approximation for determining the time required for a contactor section to reach steady-state is based on Newton's mixing vessel model in which a contactor section is represented as an ideal mixing tank with an inlet and an outlet and their respective concentrations, C_i and C_e (see Figure S1).[1] The system is regarded as having reached steady-state when the outlet concentration becomes approximately constant with respect to time, to be determined by a set criteria (*i.e.* the concentration reaches within 5% of the steady-state value). The model described relies on empirical data for holdup, $V_{section}$, and throughput $q_{section}$. It is important to note that the model assumes ideal mixing (where the concentration within the contactor, $C_{section}$, is equal to C_e) within the contactors and does not take into account chemical kinetics, which may dampen the transient behavior if the mass transfer rates between the phases are slow when compared with the flow rates.

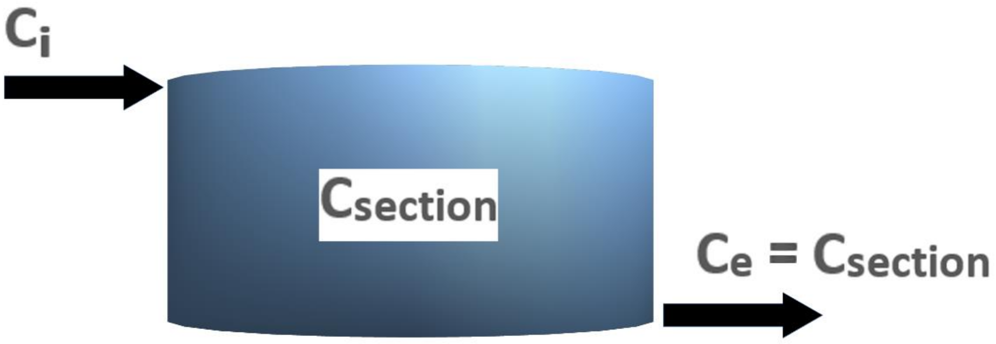

Figure S1. Control volume model showing the concentrations of the flow into the control volume as well as the flow out, which is equal to the concentration inside.

Conservation of volume requires that for a mixing vessel containing a constant volume, the volume that enters the vessel, ΔV_i, must equal the volume leaving the vessel, ΔV_e, which can be defined as

$$\Delta V_i = \Delta V_e = q_{section} * \Delta t.$$

Likewise, the concentrations of the control volume, feed, and effluent may be related using conservation of mass:

$$V_{section} * \Delta C_{section} = C_i * \Delta V_i - C_e * \Delta V_e$$

If both sides are divided by Δt and ideal mixing is assumed, taking the limit as $\Delta t \rightarrow 0$ the following differential equation is produced

$$V_{section} * \dot{C}_{section} = -q_{section} * (C_{section} - C_i).$$

The differential equation is easily solved to be

$$C_{section}(t) = \left(C_{section,o} - C_i\right)e^{-\frac{q_{section}}{V_{section}}t} + C_i ,$$

where $C_{section,o}$ is the initial condition for the concentration of the mixing tank. The response of the section concentration to a perturbation in the input concentration is shown in Figure S2 where the settling time is dependent on the time constant

$$\tau = \frac{V_{section}}{q_{section}},$$

where $t = 3\tau$ results in a concentration within 5% of steady-state.

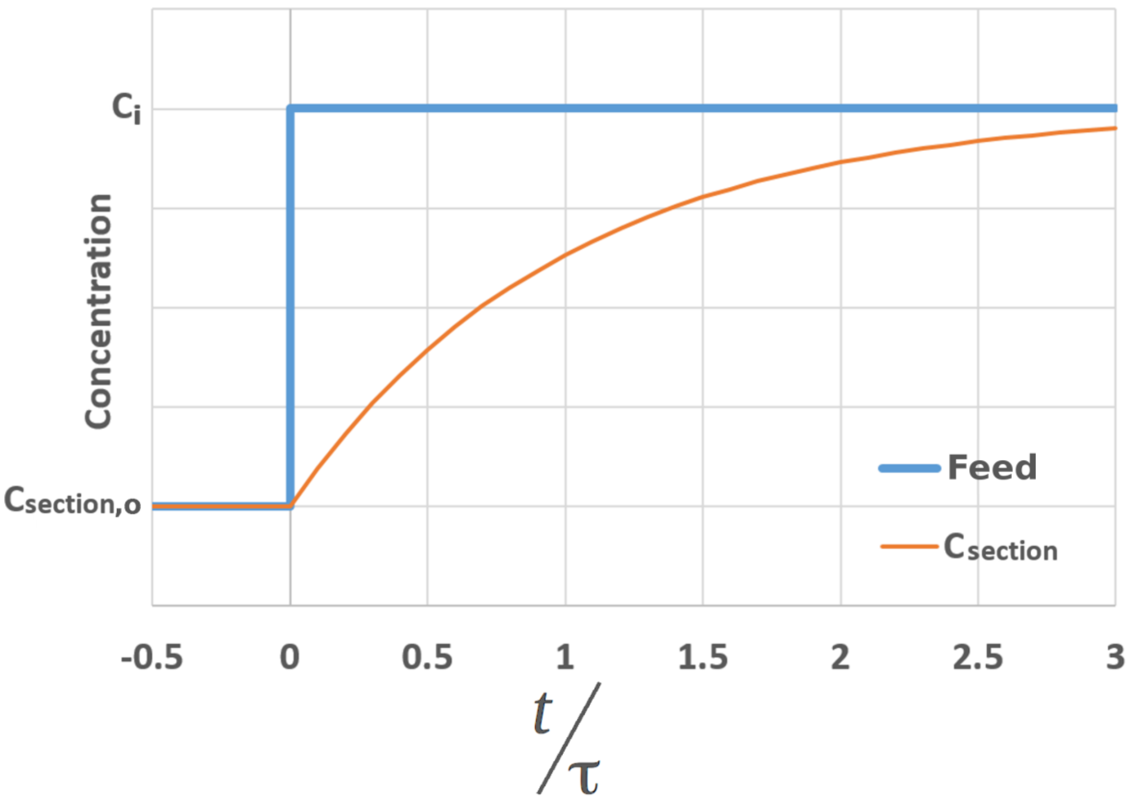

Figure S2. Concentration profile as a function of time

References

1 Stewart, J. *Differential Equations: Mixing Problems in Single Variable Calculus: Early Transcendentals, 8th ed* Vol. 2 9 (Cengage Learning, 2016).
